# Association between serum HER2/ErbB2 levels and coronary artery disease: a case–control study

**DOI:** 10.1186/s12967-020-02292-1

**Published:** 2020-03-11

**Authors:** Wen Jian, Chun-Mei Wei, Jia-Hui Guan, Chang-Hua Mo, Yu-Tao Xu, Wen-Bo Zheng, Lang Li, Chun Gui

**Affiliations:** 1 Department of Cardiology, The First Affiliated Hospital of Guangxi Medical University, 06 Shuangyong Road, Nanning, 530021 Guangxi People’s Republic of China; 2 Guangxi Key Laboratory Base of Precision Medicine in Cardio-Cerebrovascular Diseases Control and Prevention, Nanning, 530021 Guangxi People’s Republic of China; 3 Guangxi Clinical Research Center for Cardio-Cerebrovascular Diseases, Nanning, 530021 Guangxi People’s Republic of China; 4 Department of Respiratory Medicine, The First Affiliated Hospital of Guangxi Medical University, Nanning, 530021 Guangxi People’s Republic of China

**Keywords:** Coronary artery disease, HER2/ErbB2, Risk factor

## Abstract

**Background:**

Research has associated human epidermal growth factor receptor (HER2) with glucose and lipid metabolism. However, the association between circulating HER2 levels and coronary artery disease (CAD) remains to be elucidated.

**Methods:**

We performed a case–control study with 435 participants (237 CAD patients and 198 controls) who underwent diagnostic coronary angiography from September 2018 to October 2019. Adjusted odds ratios (ORs) and 95% confidence intervals (CIs) for CAD were calculated with multiple logistic regression models after adjustment for confounders.

**Results:**

Overall, increased serum HER2 levels were independently associated with the presence of CAD (OR per 1-standard deviation (SD) increase: 1.438, 95% CI 1.13–1.83; P = 0.003) and the number of stenotic vessels (OR per 1-SD increase: 1.399, 95% CI 1.15–1.71; P = 0.001). In the subgroup analysis, a significant interaction of HER2 with body mass index (BMI) on the presence of CAD was observed (adjusted interaction P = 0.046). Increased serum HER2 levels were strongly associated with the presence of CAD in participants with BMI ≥ 25 kg/m^2^ (OR per 1-SD increase: 2.143, 95% CI 1.37–3.35; P = 0.001), whereas no significant association was found in participants with BMI < 25 kg/m^2^ (OR per 1-SD increase: 1.225, 95% CI 0.90–1.67; P = 0.201).

**Conclusion:**

Elevated HER2 level is associated with an increased risk of CAD, particularly in people with obesity. This finding yields new insight into the pathological mechanisms underlying CAD, and warrants further research regarding HER2 as a preventive and therapeutic target of CAD.

## Background

Human epidermal growth factor receptor (HER) proteins are a family of receptors with tyrosine kinase catalytic activity. The HER family consists of four members, namely, HER1/ErbB1/EGFR, HER2/ErbB2, HER3/ErbB3, and HER4/ErbB4. Signaling cascades initiated by these receptors can mediate cell growth, survival, and differentiation through various pathways [1]. HER2, a 185-kDa transmembrane protein, plays a critical role in facilitating uncontrolled cell growth and tumorigenesis in various cancers [2, 3]. HER2 comprises an extracellular domain, a transmembrane domain, and an intracellular tyrosine kinase domain. Although no specific ligand has been found, HER2 can be activated upon homo- and heterodimerization with another member of the HER family. As an important signal amplifier, HER2 mediates diverse functions related to the HER family [4]. Notably, HER2 signaling was found to be significantly enhanced after proteolytic cleavage of its extracellular domain (known as HER2 shedding) [5]. As the cleaved soluble HER2 proteins can be easily measured in the bloodstream, circulating HER2 levels have relevant clinical potential.

Although important to homeostasis, abnormal regulation or production of HER2 can promote diseases [2, 3]. Intriguingly, clinical evidences have recently associated HER2 activity with glucose and lipid metabolism [6–9]. Circulating HER2 levels are found to be positively correlated with the parameters of obesity and insulin resistance [7, 8], and increased circulating HER2 levels are associated with a higher risk of developing diabetes [9]. Moreover, circulating HER2 has been identified as a causal mediator of chronic kidney disease (CKD) and can be regulated by the renin-angiotensin system [10]. These findings confirm the causal role of HER2 in various diseases besides cancer.

HER2 is abundantly expressed throughout the cardiovascular system, including by endothelial cells, vascular smooth muscle cells (SMCs), and cardiomyocytes [11–13]. Previous studies found that the HER family, particularly EGFR and HER2, contribute to the pathogenesis of vascular remodeling and atherosclerosis, such as oxidative stress, macrophage infiltration, and SMC proliferation and migration [13–16]. However, in the clinical setting, the relation between circulating HER2 levels and coronary artery disease (CAD) remains to be elucidated. With the aforementioned background, this case–control study aimed to investigate whether circulating HER2 levels are associated with CAD risk.

## Methods

### Study population

We selected consecutive symptomatic patients without known CAD who presented to the outpatient department and were scheduled for diagnostic coronary angiography in the First Affiliated Hospital of Guangxi Medical University, China, from September 2018 to October 2019. The following exclusion criteria were applied: (a) acute myocardial infarction, acute decompensated heart failure, tachyarrhythmia and severe comorbidities; (b) valvular or congenital heart disease, severe inflammatory conditions, autoimmune diseases, tumor, and end-stage renal disease; (c) missing samples for biomarker measurement due to numerous reasons. Based on the angiographic results recorded by experienced angiographers, patients with stenosis ≥ 50% of at least one epicardial vessel were enrolled as CAD group. In the meantime, patients without any visible lesion were chosen as control group, while those with mild coronary stenosis (< 50%) were excluded from this study. A total of 435 patients (CAD group, n = 237; control group, n = 198) were eligible and included for the final analysis. The study was approved by the Human Research Ethics Committee of the First Affiliated Hospital of Guangxi Medical University, China. All patients provided written informed consents.

### Data collection and related definition

For each participant, the following baseline characteristics were collected at admission: age, gender, weight, height, smoking habit, history of hypertension, history of diabetes, history of hyperlipemia, medication history, blood pressure (systolic blood pressure, diastolic blood pressure), blood glucose (fasting glucose, hemoglobin A1c [HbA1c]), blood lipids (total cholesterol, low-density lipoprotein cholesterol [LDL-C], triglycerides, high-density lipoprotein cholesterol [HDL-C]), and serum creatinine.

Former smoker was defined as smoking cessation for at least 6 months. Body mass index (BMI) was defined as the body mass divided by the square of the body height (kg/m^2^). Diabetes mellitus was defined as either a diagnosis based on the American Diabetes Association guidelines [17] or a history of taking antidiabetic medications. Hypertension was defined as the presence of systolic blood pressure > 140 mmHg, or diastolic blood pressure > 90 mmHg, or a history of taking antihypertensive medications. Dyslipidemia was defined as total cholesterol ≥ 5.2 mmol/L, LDL-C ≥ 3.4 mmol/L, or triglycerides ≥ 1.7 mmol/L, or a history of hyperlipemia with the use of cholesterol-lowering medication. Estimated glomerular filtration rate (eGFR) was calculated with the CKD-EPI equation [18].

### HER2 measurement

Overnight fasting blood samples were collected at admission for all participants. The samples were drawn into the dry tube without anticoagulant for about half an hour at room temperature, and then were processed into serum after being centrifuged at 2600 g for 10 min and stored at −80 °C. Serum HER2 levels were measured with an enzyme-linked immunosorbent assay kits (R&D Systems, Minneapolis, MN, USA) following the manufacturer’s instructions.

### Statistical analysis

Continuous variables are presented as mean ± standard deviation (SD) (for normal distributions) or median (interquartile range) (for skewed distributions), and were compared using the Student t test or the Mann–Whitney U test, as appropriate. Categorical variables are presented as percentage of patients and compared by Chi square or Fisher exact test. Spearman correlation and multivariate linear regression model were used to test the relation between clinical variables and serum HER2 levels. Univariable and multivariable binary logistic regression were used to investigate the association of HER2 levels with the presence of CAD. Multivariable ordinal logistic regression model was established to evaluate the association of HER2 with the number of stenotic coronary vessels. Confounders were selected by clinical judgment regarding the conventional CAD risk factor. Model 1 was unadjusted. Model 2 was adjusted for age and sex. Model 3 was adjusted for age, sex, BMI, smoking habit, hypertension, diabetes, dyslipidemia, and eGFR. For each model, the z scores of HER2 were included as a continuous variable to calculate the odds ratios (ORs) per 1-SD increase with 95% confidence intervals (CIs). Then, we categorized all participants into quartiles according to HER2 concentrations, and calculated ORs with 95% CIs using the lowest quartile as the reference. To assess the interaction effects of HER2 levels and conventional risk factors on the presence of CAD, interaction terms “HER2 × relevant variables” were introduced in the final multivariate model. A two-tailed P value < 0.05 was regarded statistically significant. All analyses were done using SPSS, version 22.0 and MedCalc, version 18.2.1.

## Results

### Baseline characteristics

The clinical characteristics of the study participants are detailed in Table 1. Compared with the controls, CAD patients were older, predominantly male, and had a higher frequency of smoking, hypertension, and diabetes. In addition, CAD patients had higher levels of HbA1c, fasting glucose, triglycerides, and serum creatinine, but lower levels of HDL-C, and eGFR. No significant differences in BMI, total cholesterol, and LDL-C were found between CAD patients and controls. CAD patients had a higher frequency of dyslipidemia, although this was not statistically significant (P = 0.067).

**Table 1:** Baseline data

	CAD group (n = 237)	Control group (n = 198)	*P* value
Age (years)	61 ± 10	57 ± 10	< 0.001
Male gender	176 (74%)	116 (59%)	0.001
BMI (kg/m^2^)	24.48 ± 3.31	24.25 ± 3.56	0.475
Smoking habit			0.001
Never smoker	135 (57%)	146 (74%)	
Former smoker	16 (7%)	10 (5%)	
Current smoker	86 (36%)	42 (21%)	
Hypertension	155 (65%)	107 (54%)	0.016
Diabetes	77 (33%)	27 (14%)	< 0.001
Dyslipidemia	159 (67%)	116 (59%)	0.067
HbA1_c_ (%)	6.1 (5.7–6.8)	5.9 (5.6–6.2)	< 0.001
Fasting glucose (mmol/L)	4.9 (4.5–5.8)	4.7 (4.3–5.1)	0.001
Total cholesterol (mmol/L)	4.73 ± 1.17	4.60 ± 1.05	0.259
Triglycerides (mmol/L)	1.55 (1.04–2.32)	1.27 (0.85–1.82)	< 0.001
LDL-C (mmol/L)	2.86 ± 1.01	2.74 ± 0.87	0.180
HDL-C (mmol/L)	0.99 (0.87–1.16)	1.06 (0.91–1.25)	0.006
Serum creatinine (µ mol/L)	82 (71–95)	74 (63–83)	< 0.001
eGFR (mL/min/1.73 m^2^)	84 (72–95)	93 (83–99)	< 0.001
HER2 (pg/mL)	4851 ± 1045	4596 ± 781	0.004

Continuous variables are presented as mean ± SD (for normal distributions) or median (interquartile range) (for skewed distributions). Categorical variables are presented as n (%)

*BMI* body mass index, *eGFR* estimated glomerular filtration rate, *HbA1*_*c*_ hemoglobin A1c, *HDL-C* high-density lipoprotein cholesterol, *HER2* human epidermal growth factor receptor 2, *LDL-C* low-density lipoprotein cholesterol

### Association between clinical variables and serum HER2 levels

Spearman correlation analysis showed that serum HER2 levels were positively correlated with BMI (r = 0.312, P < 0.001), HbA1c (r = 0.104, P = 0.036), total cholesterol (r = 0.268, P < 0.001), triglycerides (r = 0.276, P < 0.001), and LDL-C (r = 0.247, P < 0.001), but negatively correlated with age (r = − 0.246, P < 0.001). No correlations were found between serum HER2 levels and fasting glucose, HDL-C, and eGFR. To examine the independent determinants of HER2 variability, we performed multivariate linear regression analysis with HER2 as a dependent variable. In the entire study population, BMI presented the strongest independent association with HER2 levels (Table 2). Other independent positive determinants of HER2 variability were LDL-C and triglycerides, whereas age was negatively correlated with HER2 levels. Then, we also performed multivariate linear regression in CAD group and control group, respectively. Similarly, there were independent associations of BMI and triglycerides with HER2 levels in both CAD and control group (Table 2). Age and LDL-C were independently correlated with HER2 levels in control group, whereas they lost correlation in CAD group. Notably, in CAD group, hypertension was also a contributor to HER2 variability. All other tested associations were not significant (Table 2).

**Table 2:** Association between clinical variables and serum HER2 levels

	Entire study population			CAD group			Control group		
	β coefficient	95% CI	P	β coefficient	95% CI	P	β coefficient	95% CI	P
Age	− 0.158	− 0.272 to − 0.044	*0.007*	− 0.112	− 0.298 to 0.075	0.240	− 0.257	− 0.395 to − 0.119	*< 0.001*
Male gender	0.048	− 0.171 to 0.268	0.665	− 0.202	− 0.559 to 0.155	0.265	0.205	− 0.059 to 0.470	0.127
BMI	0.212	0.114 to 0.310	*< 0.001*	0.335	0.183 to 0.487	*< 0.001*	0.133	0.008 to 0.257	*0.037*
Current smoking	0.164	− 0.059 to 0.387	0.149	0.287	− 0.032 to 0.606	0.078	− 0.088	− 0.392 to 0.215	0.567
Hypertension	− 0.174	− 0.360 to 0.012	0.067	− 0.297	− 0.588 to − 0.007	*0.045*	− 0.096	− 0.324 to 0.132	0.408
HbA1c	0.086	− 0.005 to 0.176	0.064	0.032	− 0.090 to 0.154	0.607	0.093	− 0.053 to 0.238	0.210
LDL-C	0.180	0.090 to 0.269	*< 0.001*	0.106	− 0.022 to 0.234	0.106	0.218	0.085 to 0.351	*0.001*
HDL-C	0.082	− 0.010 to 0.175	0.080	0.013	− 0.106 to 0.132	0.835	0.172	− 0.007 to 0.351	0.060
Triglycerides	0.184	0.092 to 0.276	*< 0.001*	0.175	0.049 to 0.301	*0.007*	0.197	0.053 to 0.341	*0.008*
eGFR	− 0.021	− 0.126 to 0.085	0.697	0.067	− 0.081 to 0.216	0.372	− 0.084	− 0.235 to 0.067	0.271

Multivariate linear regression analysis with HER2 levels as a dependent variable in the entire study population, CAD group, and control group, respectively. The β coefficient for the continuous variables is expressed as per 1-SD increase to allow comparison among effects

*BMI* body mass index, *CI* confidence interval, *eGFR* estimated glomerular filtration rate, *HbA1c* hemoglobin A1c, *HDL-C* high-density lipoprotein cholesterol, *LDL-C* low-density lipoprotein cholesterol

### Association between HER2 levels and the presence of CAD

**Fig. 1:**
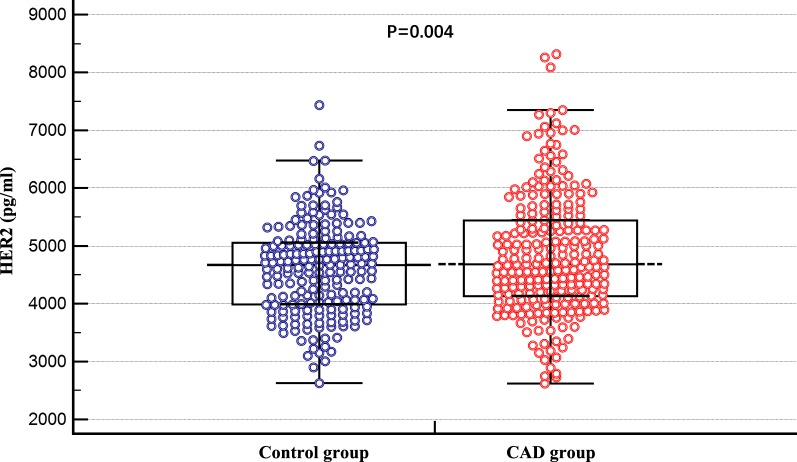
Differences of the serum HER2 levels between CAD and control group

Serum HER2 levels were significantly higher in CAD patients than in controls (4851 ± 1045 vs. 4596 ± 781 pg/mL, P = 0.004; Fig. 1). As shown in Table 3, each 1-SD increase in serum HER2 levels was associated with a 1.323-fold (P = 0.005) increased risk of CAD in the crude model. The risk remained strongly significant after adjustment of age and sex in model 2 (OR per 1-SD increase: 1.533, 95% CI = 1.23–1.91; P < 0.001) and after full adjustment in model 3 (OR per 1-SD increase: 1.438, 95% CI = 1.13–1.83; P = 0.003). When HER2 levels were analyzed as an ordinal variable, the risk of CAD was 2.365-fold (P = 0.009) higher in the highest quartile than that in the lowest quartile (model 3, Table 3). However, the 2nd and 3rd quartiles did not show a significantly high risk (P = 0.068 and P = 0.653, respectively).

**Table 3:** Binary logistic regression analyses for the association of serum HER2 levels with the presence of CAD

	Model 1		Model 2		Model 3	
	OR (95% CI)	P value	OR (95% CI)	P value	OR (95% CI)	P value
Each 1-SD increase	1.323 (1.086–1.612)	0.005	1.533 (1.231–1.908)	< 0.001	1.438 (1.130–1.830)	0.003
Quartiles of HER2						
Quartile 1	1	–	1	–	1	–
Quartile 2	1.476 (0.863–2.524)	0.155	1.578 (0.892–2.791)	0.117	1.757 (0.960–3.216)	0.068
Quartile 3	0.787 (0.461–1.343)	0.379	0.929 (0.527–1.636)	0.798	0.869 (0.471–1.602)	0.653
Quartile 4	2.104 (1.215–3.644)	0.008	2.866 (1.579–5.202)	0.001	2.365 (1.242–4.503)	0.009

Model 1 was the unadjusted model; Model 2 was adjusted for age and sex; Model 3 was adjusted for age, sex, BMI, smoking habit, hypertension, diabetes, dyslipidemia, and eGFR. CI, confidence interval; HER2, human epidermal growth factor receptor 2; OR, odds ratio; SD, standard deviation

### Association between HER2 levels and CAD severity

**Fig. 2:**
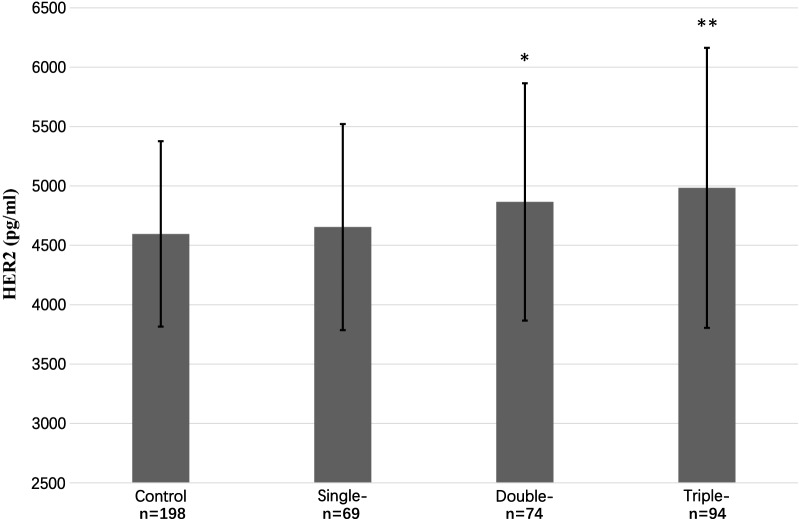
Serum HER2 levels stratified by the number of stenotic vessels. Error bars represent the standard deviation of the means. *P<0.05 vs Control; **P<0.01 vs Control

CAD patients were categorized into single-, double-, and triple-vessel disease subgroups based on the number of stenotic vessels. Of the 237 CAD patients, 69 had single-vessel disease, 74 had double-vessel disease, and 94 had triple-vessel disease. Serum HER2 levels were positively correlated with the number of stenotic vessels (Spearman correlation, r = 0.126, P = 0.009). Notably, while patients with triple-vessel disease had the highest levels of HER2, no significant difference was found between single-vessel disease and controls (4654 ± 868 vs. 4596 ± 781 pg/mL, P = 0.605, Fig. 2). In addition, patients with multi-vessel disease (≥ 2 vessels) had significantly higher HER2 levels than single-vessel disease (4932 ± 1102 vs. 4654 ± 868 pg/mL, P = 0.041). In a multivariable ordinal logistic regression model, increased serum HER2 levels were independently associated with the number of stenotic vessels (OR per 1-SD increase: 1.399, 95% CI 1.15–1.71; P = 0.001; Table 4).

**Table 4:** Ordinal logistic regression analyses for the association of clinical variables with the number of stenotic vessels

	Multivariate	
	OR (95% CI)	P value
Age, per 1 year	1.046 (1.022–1.070)	< 0.001
Male gender	1.777 (1.110–2.846)	0.017
BMI, per 1 kg/m^2^	0.988 (0.931–1.048)	0.684
Smoking habit		
Former smoker	1.303 (0.589–2.886)	0.513
Current smoker	2.441 (1.525–3.908)	< 0.001
Hypertension	1.085 (0.738–1.596)	0.678
Diabetes	2.580 (1.670–3.988)	< 0.001
Dyslipidemia	1.594 (1.063–2.389)	0.024
eGFR, per 1 mL/min/1.73 m^2^	0.983 (0.971–0.996)	0.011
HER2, per 1 SD	1.399 (1.145–1.710)	0.001

The multivariable model included age, sex, BMI, smoking habit, hypertension, diabetes, dyslipidemia, eGFR, and HER2

*BMI* body mass index, *CI* confidence interval, *eGFR* estimated glomerular filtration rate, *HER2* human epidermal growth factor receptor 2, *OR* odds ratio, *SD* standard deviation

### Interaction of HER2 with conventional risk factors on the presence of CAD

**Fig. 3:**
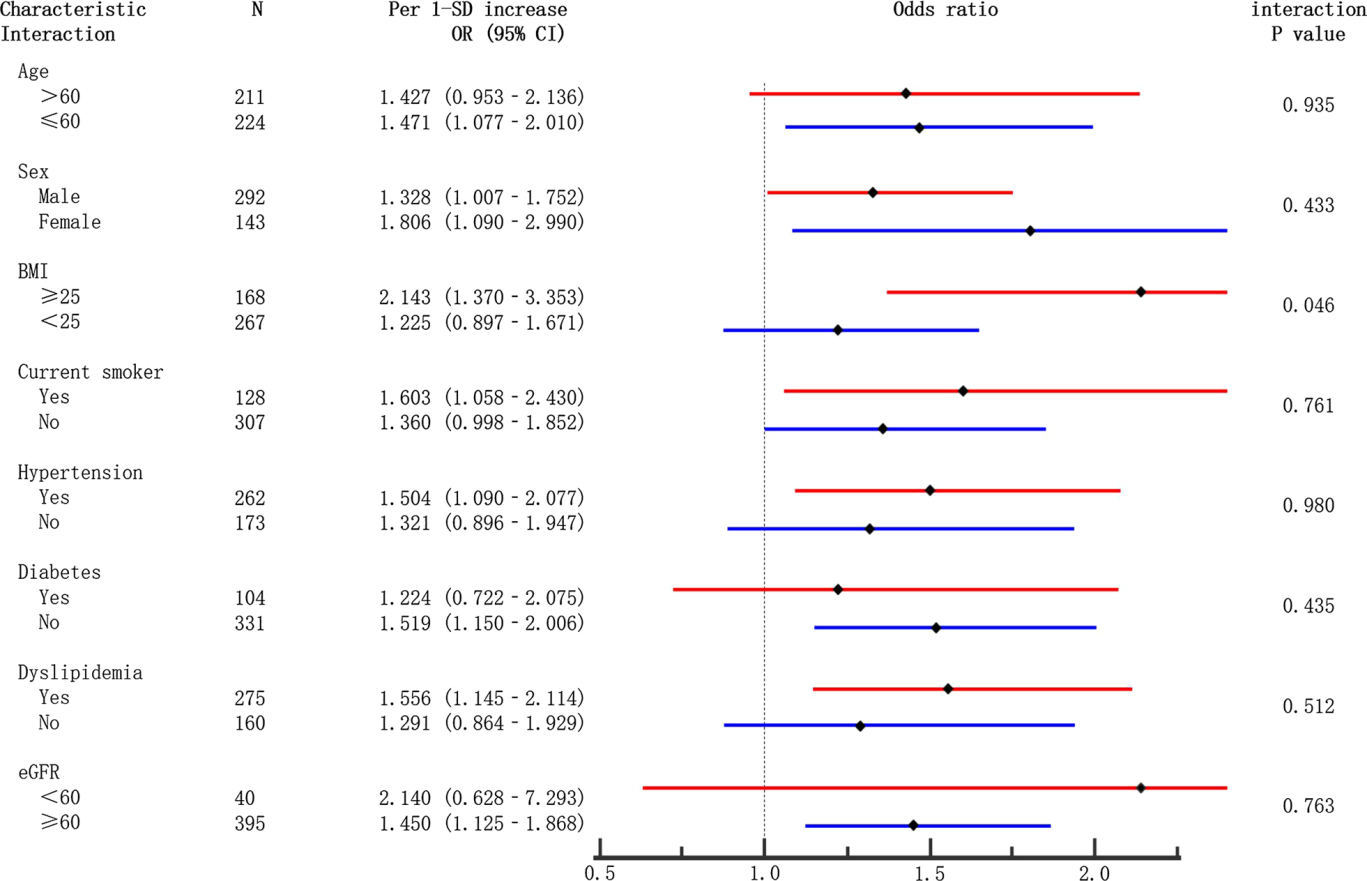
Association of HER2 levels with CAD risk in the subgroup analysis. ORs and their 95% CIs were adjusted for age, sex, BMI, smoking habit, hypertension, diabetes, dyslipidemia, and eGFR. Subgroups were as follows: age (>60, ≤ 60), sex (male, female), BMI (BMI ≥ 25, BMI<25), current smoker, hypertension, diabetes, dyslipidemia, and eGFR (eGFR<60, eGFR ≥ 60)

Figure 3 shows a significant interaction of HER2 with BMI on the presence of CAD (adjusted interaction P = 0.046). After full adjustment, increased serum HER2 levels were strongly associated with the presence of CAD in participants with BMI ≥ 25 kg/m^2^ (OR per 1-SD increase: 2.143, 95% CI 1.37–3.35; P = 0.001), but no significant association was found in participants with BMI< 25 kg/m^2^ (OR per 1-SD increase: 1.225, 95% CI 0.90–1.67; P = 0.201). Similar results were also observed when HER2 levels were analyzed as an ordinal variable. The adjusted OR for the highest versus the lowest quartile of HER2 was 5.099 (95% CI 1.52–17.06; P = 0.008) in participants with BMI ≥ 25 kg/m^2^ and was 1.731 (95% CI 0.74–4.05; P = 0.206) in participants with BMI < 25 kg/m^2^. However, no interaction was observed between HER2 and age (> 60, ≤ 60), sex (male, female), current smoking, hypertension, diabetes, dyslipidemia, and eGFR (eGFR < 60, eGFR ≥ 60) on the presence of CAD (Fig. 3).

## Discussion

To the best of our knowledge, this study is the first to report that an elevated HER2 level is associated with an increased risk of CAD, particularly in people with obesity. This association remained significant even after adjusting for conventional CAD risk factors.

The HER family has been previously implicated in the pathogenesis of atherosclerosis in various animal models. EGFR, which is the most characterized member, contributes to cardiovascular disorders by causing an increase in oxidative stress, macrophage infiltration, release of proinflammatory cytokines, and transformation of SMC phenotype by a mechanism referred to as “transactivation” [13, 15]. EGFR can be transactivated by various factors, including endothelin-1, angiotensin II, norepinephrine, prorenin, and thrombin, which interact with other cellular communication networks in the context of atherosclerotic pathologies [19, 20]. Many experimental studies have shown that the inhibition of EGFR signaling can significantly attenuate atherosclerosis in mice models [16, 21, 22]. As a preferred heterodimeric partner, HER2 not only provides a platform which amplifies EGFR biological responses, but can also be activated through phosphorylation of its intracellular tyrosine kinase domain by EGFR; upon phosphorylation, it triggers downstream signaling [13, 23]. Likewise, increased HER2 signaling can stimulate SMC growth, proliferation, and migration independently of EGFR downstream pathways [13]. Meanwhile, the HER2 shedding process, which is attributed to various zinc-containing metalloproteases, including matrix metallo-protease (MMP) families, can increase the tyrosine kinase activity and amplify HER2 signaling [5]. MMP families are well known to degrade structural proteins of the extracellular matrix, for example, the collagenous matrix in the fibrous cap and adjacent shoulder regions, and play critical roles in the instability of plaques [24]. Intriguingly, enhanced HER2 signaling can, in turn, promote the transcription of MMP families, thus probably creating a vicious cycle in the setting of atherosclerosis [25]. However, whether HER2 plays a role in other pathological mechanisms underlying CAD remains to be defined.

Emerging evidences suggest a close relationship between CAD and cancer, which is attributed to shared risk factors such as obesity, diabetes, hypertension, and dyslipidemia [26, 27]. However, CAD and cancer may also overlap with some potential biological pathways which have not been fully understood. Together with our findings, we assume that HER2, an oncogenic marker, is more likely to be a mediator of CAD, and people with elevated HER2 levels may possess a higher risk of CAD. Indeed, there is no evidence of reverse causation between circulating HER2 levels and CAD in the current literature. In vivo, sustained hypoxia has no significant effect on HER2 expression by endothelial cells and SMCs [28, 29]. In both the early and decompensated stages of heart failure, myocardial HER2 expression has been found to be significantly decreased [30, 31]. Similarly, in biopsies from CAD patients, HER2 expression was markedly downregulated in hypoxic samples compared with that in normoxic samples [32]. Intriguingly, we found that HER2 levels in the bloodstream are higher in CAD patients than in controls, which indicates that circulating HER2 level represents the systemic activity of HER2 signaling rather than its local expression.

HER2 is essential throughout the cardiovascular system [11–13], and its extracellular domain shedding process make it highly expressed in the bloodstream [5]. Of note, our data showed that the difference in circulating HER2 levels between CAD and control groups was not strikingly huge. One possible explanation is that there is a tremendous interindividual variability in circulating HER2 levels, and it may probably have pathological relevance only when HER2 reaches a certain high level. Muhammad et al. [9] performed a population-based cohort study, and they found that whereas participants in the highest HER2 quartile had a significant higher risk of developing diabetes than those in the lowest quartile, the 2nd and 3rd quartiles did not exhibit a higher risk after adjustment for confounders. Similarly, this phenomenon was also observed in our case–control study when we categorized all participants into quartiles according to HER2 concentrations. Given the fact that CAD is a multifactorial disease influenced by both environmental and genetic factors [33], the slightly elevated HER2 levels may not predispose people to CAD. Thus, a huge range of HER2 levels overlapped between CAD and control groups in our study. Further large cohort studies are needed to verify our findings and explore the optimal risk threshold of HER2 levels.

It is well known that patients with CKD more frequently experience cardiovascular disease than those without [34]. Considering that HER2 is a causal mediator of CKD [10], HER2 may indirectly take an influence on CAD partly through CKD. Thus, adjusting baseline eGFR in our regression models may probably underestimate the risk effect of HER2. Even so, we still observed an independent association between circulating HER2 levels and CAD after full adjustment in this study, which confirms a potential role for HER2 in the progress of CAD.

Obesity, which is characterized by dyslipidemia, increased blood glucose, and abnormal levels of hormone-like adipokines, mediates chronic inflammation that promotes diseases [35, 36]. MEMON et al. [8] reported that there was an independent association between HER2 levels and hyperglycemia in Swedish people. Fernandez-Real et al. [7] showed that fasting triglyceride was an independent factor which contributed to 10–11% of serum HER2 variance in a cross sectional study. They also found that serum HER2 levels could significantly decrease after weight loss in obese subjects. Similarly, in our multivariate linear regression analysis, we observed an independent association of BMI, LDL-C and triglycerides with HER2 levels, suggesting a close relation of HER2 with metabolism. Indeed, emerging evidences show that HER2 signaling is associated with obesity and the lipid-related microenvironment, and plays an important role in adipose differentiation [6, 37]. Intriguingly, our study found an interaction between HER2 levels and BMI on the presence of CAD, indicating that HER2 signaling may significantly contribute to the progress of atherosclerosis when coupled with other factors, such as obesity. Experimental studies found that the molecules governing lipid metabolism can significantly interact with HER2 signaling, which alters the behavior of various cell types in multiple pathological conditions [6]. For instance, leptin, an important proinflammatory adipokine, has been found to cause vascular dysfunction by increasing HER2 activity in the arterial wall [13]. In turn, HER2 signaling can enhance the transcriptional activation of leptin, which creates a feedback loop [38]. Thus, taken together, the synergistic effect of obesity and HER2 may provide new insight into the pathological mechanisms underlying CAD.

Currently, tyrosine kinase inhibitors have been widely implicated in cancers [39, 40]. On the other hand, a growing evidence supports the notion that HER2 inhibitors are a potential treatment for diabetes [9, 41], obesity [37], and kidney disease [10, 42]. Our data suggest that HER2 may be a potential therapeutic target of CAD. However, given the possible cardiotoxicity of a potent anti-HER2 treatment that can lead to severe heart failure in breast cancer [43], the benefits and risks of this prospect warrant further study.

This study has several limitations that should be considered when interpreting its results. First, this was a single-center study with a small sample size. Only cases and controls that met the inclusion criteria were analyzed; thus, a certain degree of selection bias was present. Given the fact that cases and controls were not age- and gender- matched, this gap was a major limiting factor for our study. Second, considering the cross-sectional nature of this study, the observational findings must be regarded as hypothesis-generating, as they do not allow for causal inference. In addition, despite the provision of plausible explanations, we could not rule out the possibility of reverse causation. Third, although conventional CAD risk factors had been adjusted, residual unknown confounders, such as unmeasured biomarkers or mechanisms, could not be entirely ruled out. Fourth, since the controls were selected from symptomatic patients with angiographically normal coronary arteries, we could not fully exclude subjects with microcirculation disturbances or angiographically invisible lesions from the control group. Thus, it remains unknown whether these conditions influence HER2 levels. More data and verification are required in the future.

## Conclusion

Elevated HER2 level is associated with an increased risk of CAD, particularly in people with obesity. This finding yields new insight into the pathological mechanisms underlying CAD, and warrants further research regarding HER2 as a preventive and therapeutic target of CAD.

## Data Availability

The datasets used and/or analysed during the current study are available from the corresponding author on reasonable request.

## Abbreviations

BMI: Body mass index
CAD: Coronary artery disease
CKD: Chronic kidney disease
CIs: Confidence intervals
eGFR: Estimated glomerular filtration rate
HER2: Human epidermal growth factor receptor 2
HbA1c: Hemoglobin A1c; HDL-C, high-density lipoprotein cholesterol
HDL-C: High-density lipoprotein cholesterol
LDL-C: Low-density lipoprotein cholesterol
MMP: Matrix metallo-protease
ORs: Odds ratios
SD: Standard deviation
SMCs: Vascular smooth muscle cells
